# Study on the deposition behavior of sodium/sulfide elements during the operation of radiant syngas cooler in an entrained-flow gasifier

**DOI:** 10.1039/d5ra02794b

**Published:** 2025-06-25

**Authors:** Chunlei Gao, Linmin Zhang, Xudong Song, JinSheng Cao, Qinghua Guo, Guangsuo Yu

**Affiliations:** a Institute of Clean Coal Technology, East China University of Science and Technology Shanghai 200237 China; b Yankuang Energy Group Co., Ltd Shandong 273500 China; c State Key Laboratory of High-efficiency Utilization of Coal and Green Chemical Engineering, College of Chemistry and Chemical Engineering, Ningxia University Yinchuan 750021 China gsyu@nxu.edu.cn +86 21 64251312 +86 21 64252974; d Institute of Energy Materials and Devices, Structure and Function of Materials (IMD-1), Forschungszentrum Jülich GmbH 52425 Jülich Germany; e Yanzhou Coal Industry Yulin Energy and Chemical Co., Ltd Yulin 719000 China

## Abstract

As a core process in the coal chemical industry, entrained-flow gasification technology promotes clean energy conversion by efficiently producing syngas. In industrial applications, radiant syngas cooler (RSC) is usually adopted to recover high-temperature syngas and slag waste heat from the gasifier outlet to improve system heat utilization. However, the ash deposition and slagging behavior in RSC can lead to a decrease in heat transfer efficiency and limited production capacity, becoming a key bottleneck, restricting technological development. In this work, the ash slags in the critical areas (radiation screen top 25 m, 25/20/15 m around spray water, floating ash on water-cooled wall, and boiler bottom 0–1 m) in the RSC were taken as the research object, and its elemental composition, ash chemical composition, mineral evolution, and high-temperature melting, and crystallization behavior were systematically analyzed. The results showed that after rapid cooling by RSC, some carbon particles in the fly ash carried by high-temperature syngas terminated the gasification reaction due to insufficient temperature, forming residual carbon, resulting in a higher carbon content in the floating ash of the water-cooled wall. The sulfur element was primarily enriched in the middle area of the RSC (floating ash on the water-cooled wall and 15 m around spray water). Mineral evolution analysis showed that alkaline oxides (CaO, Fe_2_O_3_, Na_2_O) and acidic oxides (SiO_2_, Al_2_O_3_) in the ash slags inside the RSC underwent low-temperature eutectic reactions to form low melting point minerals, such as nepheline, anorthite, augite, and melilite. The higher content of ZnO in the ash slag at 15 m around the spray water reacted with CaO and SiO_2_ to generate the refractory mineral Ca_2_ZnSi_2_O_7_, resulting in an increase in its melting temperature. *In situ* observation showed that all the ash slag samples displayed three stages of “shrinkage–melting–flow”. Due to the formation of the refractory substance Ca_2_ZnSi_2_O_7_ in the ash slag at 15 m around the spray water during heating, its melting temperature was high and required a higher temperature to complete the flow. During the cooling and crystallization process, some interlaced rod-like crystals and a few square crystals were mainly precipitated in the slag.

## Abstract 2

This study systematically samples the ash slag in key areas of the radiant syngas cooler in the entrained-flow gasifier and explores the ash slag deposition mechanism in different spatial areas.

## Graphical abstract

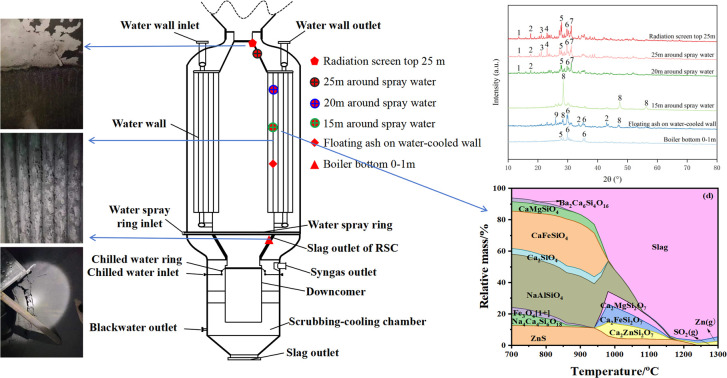

## 1. Introduction

With the increasingly severe energy crisis and environmental issues, clean coal technology has become the key direction for energy transformation. As a representative of coal-based clean conversion technology, entrained-flow gasification occupies a core position in the field of coal-based chemical synthesis and integrated gasification combined cycle (IGCC) power generation by efficiently producing syngas.^1–3^ As the entrained-flow gasifier operates at high temperature and pressure (>1200 °C, 4–6 MPa), the outlet syngas carries a large amount of sensible heat.^4–6^ It has been reported that in entrained-flow bed dry pulverized coal and coal–water slurry gasification technologies, the sensible heat of syngas can account for more than 14% and 20% of the total heat in coal, respectively.^7^ Thus, the recovery of sensible heat from high-temperature syngas can effectively enhance the overall efficiency of coal gasification. As the core equipment in entrained-flow coal gasification, the radiant syngas cooler (RSC) undertakes the dual functions of high-temperature syngas waste heat recovery and ash slag capture, and its operating efficiency is directly related to the energy consumption and economy of the system.

RSC achieves the cooling of syngas and the recovery of sensible heat through two heat transfer methods: radiation and convection.^8–10^ The high-temperature syngas entering the RSC also carries a large amount of ash slag particles, among which smaller particles adhere to the wall due to strong fluidity, while ash slag particles with larger particle sizes sink into the slag pool under the action of gravity.^11–13^ At the same time, high-temperature liquid slag (>1300 °C) flows down along the gasifier and RSC walls and is quenched in the pool. During this process, some of the slag droplets collide with the water-cooled wall under the action of airflow, and solidify into solid slag after heat exchange with the pipe wall. Therefore, during long-term operation, in addition to heat recovery, the formation and deposition of ash slag also occur in the RSC.^14–16^ It is worth noting that sulfur (S) and sodium (Na), as common components in coal, are easily volatilized at high temperature and undergo complex reactions with other ash chemical components (such as Si, Al, and Ca) to form low melting point minerals, leading to the deposition of ash slag. This not only reduces heat transfer efficiency but may also cause corrosion, slagging, and even equipment failure.^17–19^ Therefore, it is necessary to conduct in-depth research on the deposition behavior of key elements, such as S and Na, in the RSC of the entrained-flow gasifier.

This work systematically sampled key areas (radiation screen top 25 m, 25/20/15 m around spray water, floating ash on water-cooled wall, and boiler bottom 0–1 m) in the RSC, focusing on the deposition behavior at different areas in the RSC. First, the elements and ash chemical composition in different areas of the RSC were explored, the spatial distribution of element deposition and ash chemical composition was revealed, and the deposition hotspot areas were defined. Meanwhile, FactSage thermodynamic software was used to investigate the mineral evolution of ash slag at high temperature and to reveal the ash slag deposition mechanism. Finally, the high-temperature melting flow and cooling crystallization behavior of ash slag were observed *in situ* using HTSOM, reproducing the dynamic deposition process of ash slag in the RSC.

## 2. Experimental section

### 2.1 Materials

The coal used for gasification was Yulin (YL) coal from Shanxi Province. After crushing and grinding the coal sample to a particle size of <75 μm, the proximate and ultimate analysis of YL coal was conducted using an infrared rapid coal quality analyzer (5E-MACIII) and an elemental analyzer (Vario MACRO), according to national standards GB/T 212-2008 and GB/T 31391-2015, and the results are shown in Table 1. According to GB/T212-2008, the coal sample was heated to 815 °C in a muffle furnace for ashing. The specific temperature control procedure was to heat the sample from ambient temperature to 500 °C in 30 min and hold it for 30 min, then continue to heat the sample up to 815 °C at a rate of 5 °C min^−1^, and finally ash it for 2 h to completely remove organic matter. According to GB/T 1574-2007, the ash chemical composition was analyzed using an X-ray fluorescence spectrometer (PANalytical Axios; RIGAKU ZSX Priums), as shown in Table 2. The results demonstrated that the content of Fe and Ca in YL coal ash was higher, and the Si/Al ratio >2 belonged to coal with a high Si/Al ratio.

**Table 1:** Proximate and ultimate analyses of YL coal

Sample	Proximate analysis/d (wt%)			Ultimate analysis/d (wt%)				
	VM	FC	A	C	H	N	S	O^a^
YL	32.97	59.67	7.37	73.21	4.45	1.05	13.56	7.73

a Note: VM–volatile matter; FC–fixed carbon; d–dry basis; *–calculated by difference.

**Table 2:** Ash chemical composition of YL coal

Sample	Composition (wt%)									
	SiO_2_	Al_2_O_3_	Fe_2_O_3_	CaO	Na_2_O	K_2_O	MgO	SO_3_	TiO_2_	Others
YL	41.64	18.25	14.47	13.03	2.69	0.78	1.55	6.33	0.59	0.67

### 2.2 Ash fusion temperature test

**Fig. 1:**
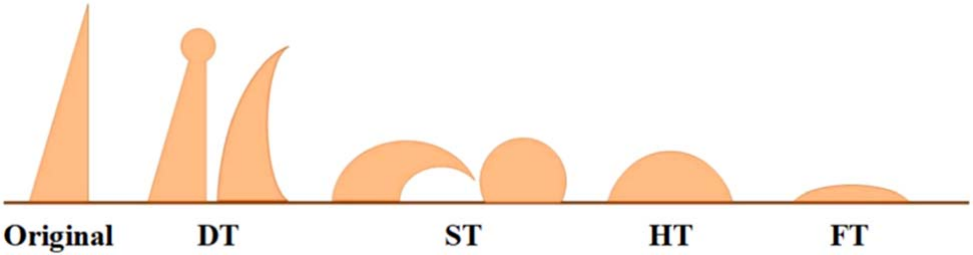
Diagram of coal ash fusion process.

The ash melting temperatures of the samples were determined based on GB/T219-2008. During the testing process, a prefabricated triangular ash cone (7 × 20 mm) was placed in a fully automated ash melting temperature analyzer (SDAF4000, Sande Technology Co., Ltd., China), and the carbon sealing method was applied to simulate a weakly reducing atmosphere. First, the temperature was raised to 900 °C at 15 °C min^−1^ and then continuously heated at a rate of 5 °C min^−1^ until the test was terminated. The deformation temperature (DT), softening temperature (ST), hemispherical temperature (HT), and flow temperature (FT) were determined by real-time comparison of the variation in ash cone with the standard characteristic temperature determination criteria, as shown in Fig. 1. As indicated in Table 3, the ash melting temperature of YL coal was low, and the difference between the four characteristic temperatures was small, representative of low ash melting point coal.

**Table 3:** Ash fusion temperatures of YL coal

Sample	Ash fusion temperature (°C)			
	DT	ST	HT	FT
YL	1080	1105	1109	1114

### 2.3 Characterization of element and ash chemical composition of ash slag in different parts of the RSC

**Fig. 2:**
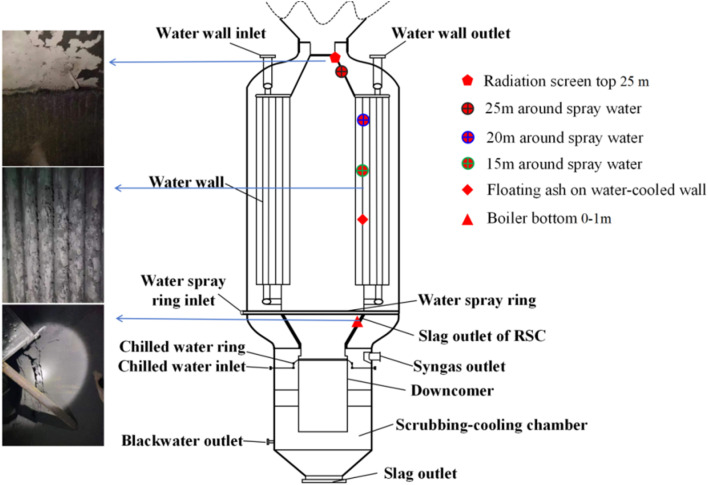
Schematic diagram of the RSC and the key areas for slag sample selection.

To investigate the elemental deposition pattern during RSC operation, ash slag samples from the key areas (radiation screen top 25 m, 25/20/15 m around spray water, floating ash on water-cooled wall, and boiler bottom 0–1 m) of the RSC were selected for elemental analysis. A schematic diagram of the RSC and the key areas for slag sample selection are shown in Fig. 2. After crushing and grinding the sample to a particle size of <75 μm, the raw materials were tested for elemental analysis using an elemental analyzer (Vario MACRO) according to the GB/T 476-2001 standard. At the same time, according to GB/T212-2008, ash slag from different key areas in the RSC was ashed, and the ash chemical composition was analyzed using an X-ray fluorescence spectrometer (PANalytic Axios; RIGAKU ZSX Priums).

### 2.4 X-ray powder diffractometer analysis

A D8 advance X-ray powder diffractometer (XRD) from Bruker-AXS, Germany, was employed to analyze the ash slag mineral composition in different key areas of the RSC. The samples were ground to less than 75 μm before the test. The test conditions were as follows: Cu target, tube voltage 40 kV, tube current 40 mA, scanning rate 5° per min, step size 0.01°, and scanning range of 10–85°.

### 2.5 FactSage thermodynamic calculations

FactSage 8.3 thermodynamics software has a powerful database in the field of computational thermochemistry, which can achieve multiphase equilibrium calculations under various constraint conditions.^20–23^ In this work, the FactSage 8.2 thermodynamics software platform was used to perform multivariate multiphase equilibrium calculations on the phase transition behavior of ash slag at high temperatures. The FToxid database was selected for calculations, and the mineral transformations and solid–liquid ratios of the samples were calculated in the Equilib module for the temperature range of 700–1300 °C with a calculation step size of 50 °C.

### 2.6 Observation of *in situ* melting and crystallization behavior using HTSOM

**Fig. 3:**
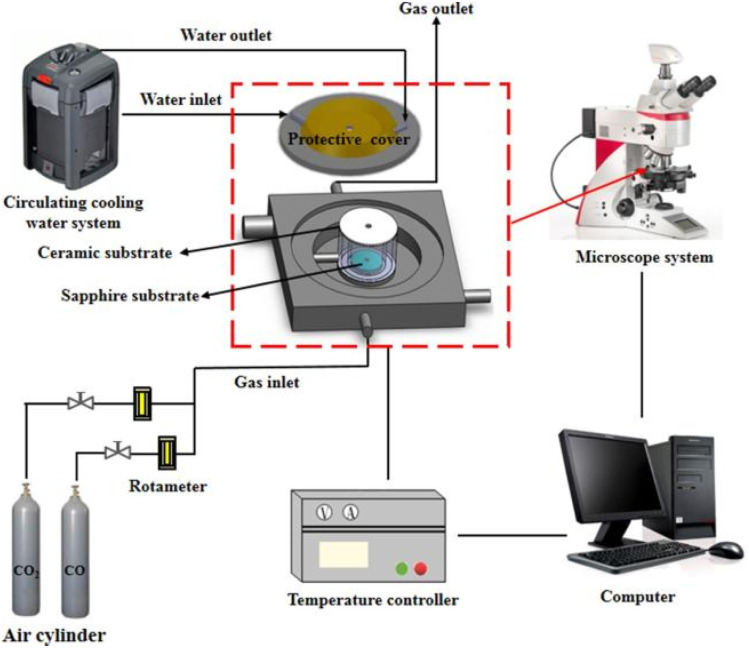
Schematic diagram of HTSOM.

**Fig. 4:**
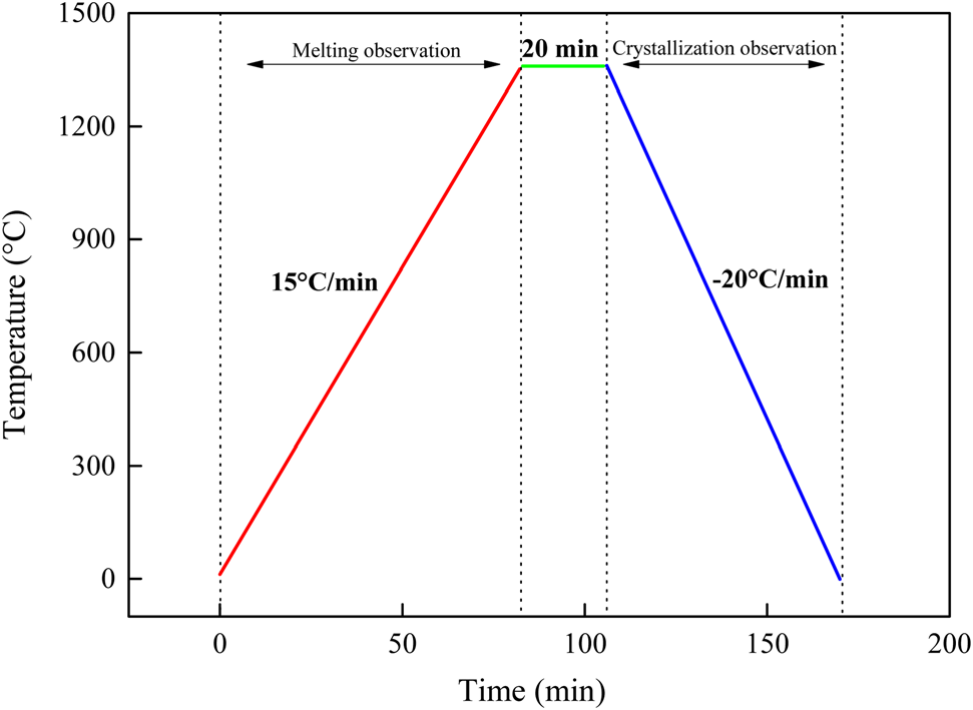
Temperature control procedure of HTSOM test.

The high-temperature melting and crystallization behavior of ash slag were observed *in situ* using a high-temperature stage coupled with an optical microscope system (HTSOM).^24–27^ The experimental system consists of a high-temperature hot stage TS1500 (Linkam, UK), a polarizing optical microscope DM2700P (Leica, Germany), a precision temperature control unit, and a data acquisition system; the schematic structure is shown in Fig. 3. The specific steps of the HTSOM experiment were as follows: about 0.1 mg of ash slag sample was uniformly spread on the sapphire surface and put into the reaction chamber and then the temperature was increased to 1300 °C at a rate of 15 °C min^−1^ under Ar atmosphere, and the melting flow behavior of the ash slag sample was recorded in real time using a microscope. When the temperature reached 1300 °C, it was left at this temperature for 20 min to ensure complete melting of the ash. Subsequently, the temperature was cooled to room temperature at 20 °C min^−1^, and the cooling crystallization behavior of the slag was recorded in real time. The temperature control procedure of the HTSOM is shown in Fig. 4.

## 3. Results and discussion

### 3.1 Analysis of elements and ash chemical composition of ash slag in different parts of the RSC

The elemental analysis of ash slag at different areas of the RSC is shown in Table 4. The results revealed a high C content in the floating ash on the water-cooled wall, indicating the presence of incompletely gasified residual carbon in the area, which can be attributed to the rapid cooling process of high-temperature syngas in the RSC. Specifically, the fly ash particles carried by the high-temperature syngas rapidly transferred heat to the water-cooled wall through radiation and convection, resulting in its temperature rapidly dropping below 800 °C. During this process, some carbon particles stopped the gasification reaction due to insufficient temperature, forming residual carbon, resulting in a high C content (6.39 wt%) in the floating ash on the water-cooled wall. Regarding the sulfur content, it was mainly distributed in the floating ash on the water-cooled wall and 15 m around the spray water, indicating that the middle region of the RSC was the hotspot for sulfur deposition. This was due to the rapid cooling of high-temperature syngas after entering the RSC, when some gaseous sulfides condensed and adsorbed onto the surface of fly ash particles, eventually leading to sulfur enrichment.

**Table 4:** Element analysis at different positions of the RSC

Sample	Elemental analysis/d (wt/%)			
	C	H	N	S
Radiation screen top 25 m	0.15	0.026	0.02	0.509
25 m around spray water	0.18	0.028	0.03	1.293
20 m around spray water	0.42	0.033	0.02	1.678
15 m around spray water	0.12	0.042	0.02	4.578
Floating ash on water-cooled wall	6.39	0.142	0.00	3.650
Boiler bottom 0–1 m	0.09	0.051	0.00	0.608

**Fig. 5:**
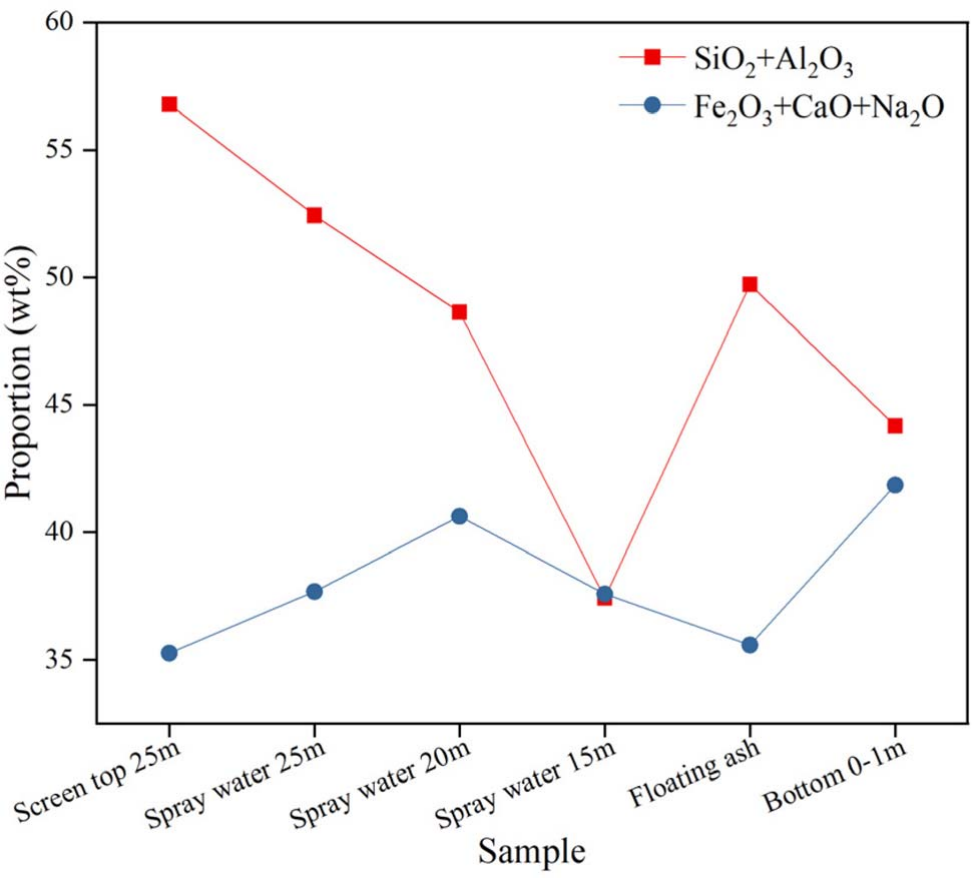
The content changes of acidic and basic oxide from top to bottom of the RSC.

**Fig. 6:**
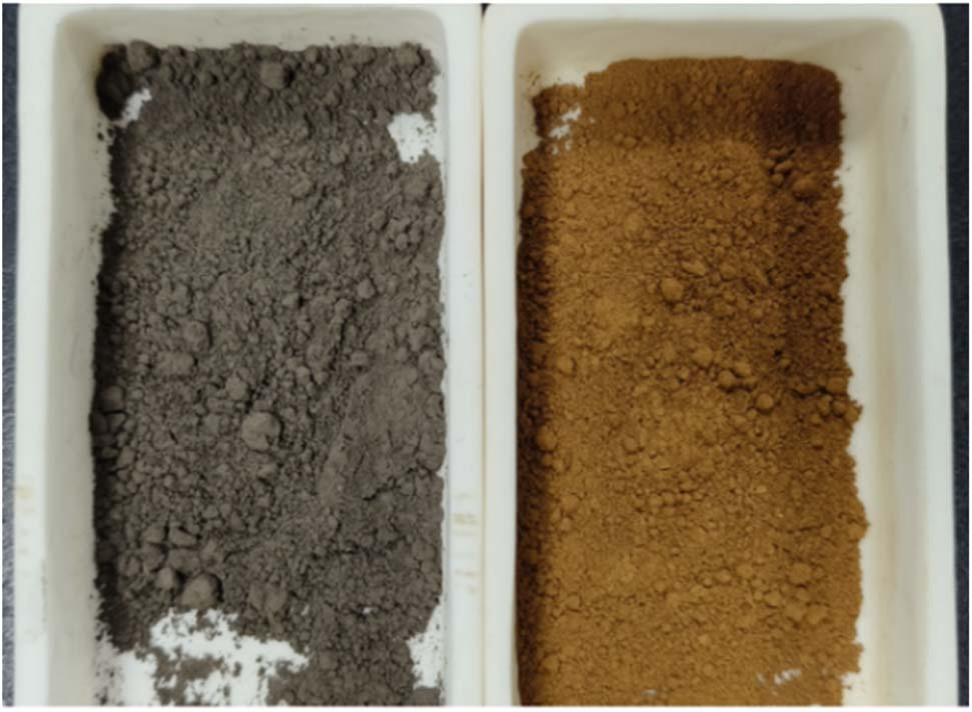
Comparison of ash slag before and after ashing.

The chemical composition of ash slag was closely related to its deposition behavior in the RSC of the gasifier. The ash chemical components of samples in different areas of the RSC after ashing are shown in Table 5. According to their properties, the oxides in the ash could be divided into acidic oxides and basic oxides. The acidic oxides were mainly SiO_2_ and Al_2_O_3_, while the basic oxides were primarily CaO, Fe_2_O_3_, and Na_2_O. The acidic oxides tend to increase the melting temperature, whereas the basic oxides tend to decrease it. Fig. 5 shows the changes in the contents of acidic oxides (SiO_2_ + Al_2_O_3_) and basic oxides (CaO + Fe_2_O_3_ + Na_2_O) from top to bottom in the RSC. It can be seen that from the top of the radiation screen at 25 m to 15 m around the spray water, the content of acidic oxides gradually decreased, while the content of basic oxides initially increased and then decreased, and the turning point occurred at 15 m around the spray water. Combined with Table 5, it can be seen that the main reason for the decrease in basic oxide content at 15 m around the spray water was due to the higher content of ZnO and SO_3_, which suggested that the ash slag deposition behavior at 15 m around the spray water may be more complicated. For the floating ash on the water-cooled wall and the ash slag at the boiler bottom 0–1 m, the acidic oxide content and the basic oxide content showed an opposite trend, and they both contained a relatively high content of SO_3_. In addition, all ash samples contained a high content of Fe_2_O_3_, which was also evident from the color change of the ash samples after ashing, which changed from gray-brown to red-brown, as shown in Fig. 6, indicating a high content of Fe.

**Table 5:** Analysis of ash chemical composition at different positions of the RSC

Sample	Ash chemical composition (wt/%)													
	SiO_2_	Al_2_O_3_	Fe_2_O_3_	CaO	SO_3_	Na_2_O	MgO	K_2_O	TiO_2_	ZnO	P_2_O_5_	BaO	SrO	Others
Radiation screen top 25 m	37.01	19.78	15.02	15.65	2.18	4.59	1.61	1.09	0.85	0.15	0.20	0.79	0.56	0.19
25 m around spray water	34.59	17.85	17.26	14.69	3.14	5.73	1.27	1.34	0.94	0.72	0.30	0.82	0.58	0.28
20 m around spray water	33.11	15.53	20.44	16.78	3.45	3.41	1.41	0.91	0.73	1.43	0.18	0.64	0.65	0.48
15 m around spray water	26.06	11.37	16.90	12.92	8.90	7.77	1.32	0.70	0.77	8.83	0.16	0.72	0.60	1.12
Floating ash on water-cooled wall	33.62	16.11	18.52	13.93	5.80	3.13	1.12	1.74	1.07	1.07	0.20	0.64	0.55	1.80
Boiler bottom 0–1 m	30.25	13.92	20.88	17.02	4.35	3.96	1.47	0.96	0.91	1.07	0.14	0.74	0.77	0.80

### 3.2 XRD analysis of ash slag from different parts of the RSC

1- clinoferrosilite (FeSiO_3_), 2-pyrite (FeS), 3-microcline (KAlSi_3_O_8_), 4-nephelin (NaAlSiO_4_), 5-anorthite (CaAlSi_2_O_8_), 6-augite (Ca(Mg,Fe)Si_2_O_6_), 7-gehlenite (Ca_2_Al_2_SiO_7_), 8-ZnS, 9s sillimanite (Al_2_SiO_5_)

**Fig. 7:**
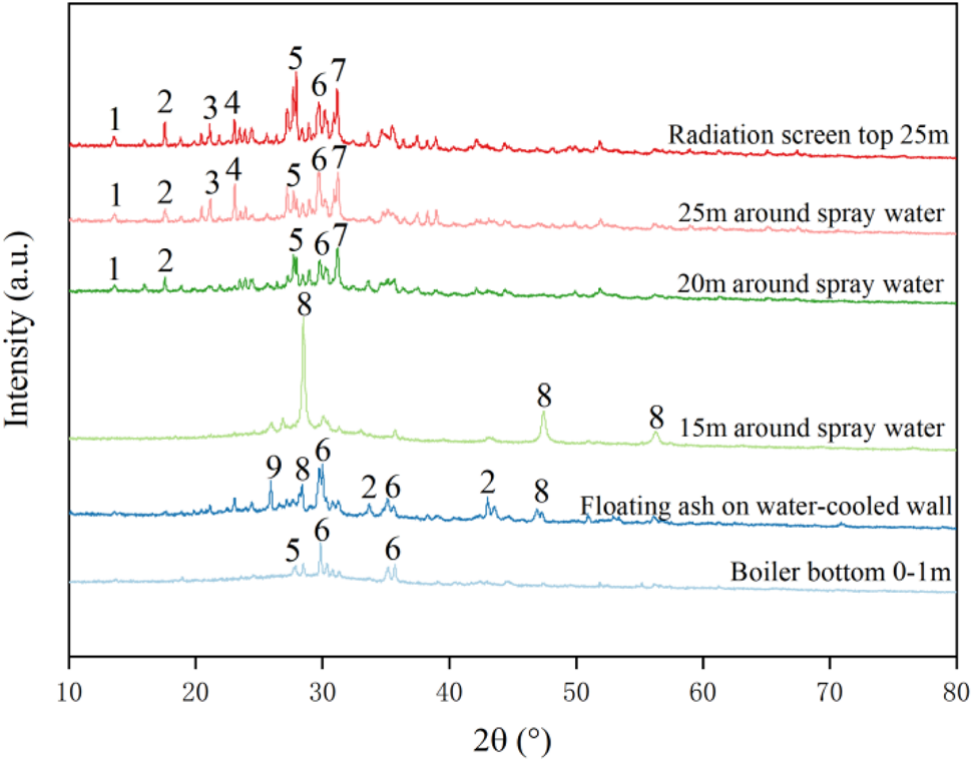
XRD patterns of ash at different positions of the RSC.

Fig. 7 demonstrates the XRD patterns of ash slag samples from different key regions in the RSC. It can be seen that the differences in chemical composition of ash slag from different regions led to certain differences in mineral types and contents. Among them, the mineral composition of ash slag at the radiation screen top 25 m and 25/20 m around the spray water was the same, mainly consisting of clinoferrosilite (FeSiO_3_), pyrite (FeS), microcline (KAlSi_3_O_8_), nepheline (NaAlSiO_4_), anorthite (CaAlSi_2_O_8_), augite (Ca(Mg,Fe)Si_2_O_6_), and gehlenite (Ca_2_Al_2_SiO_7_). Notably, the minerals of the 15 m ash slag around the spray water were all ZnS, which was related to its higher content of SO_3_ and ZnO, as shown in Table 5. Due to the highest content of alkaline components (CaO + Fe_2_O_3_), the mineral compositions of ash slag at the boiler bottom 0–1 m were mainly anorthite (CaAlSi_2_O_8_) and augite (Ca(Mg,Fe)Si_2_O_6_). The mineral compositions in the floating ash on the water-cooled wall were between the mineral composition of ash slag at the boiler bottom 0–1 m and around 15 m spray water, mainly included pyrite (FeS), augite (Ca(Mg,Fe)Si_2_O_6_), ZnS, and sillimanite (Al_2_SiO_5_). Based on the above mineral composition analysis, it was discovered that the mineral composition of ash slag samples from different regions of the RSC followed a certain spatial distribution pattern.

### 3.3 Mineral transformation analysis

**Fig. 8:**
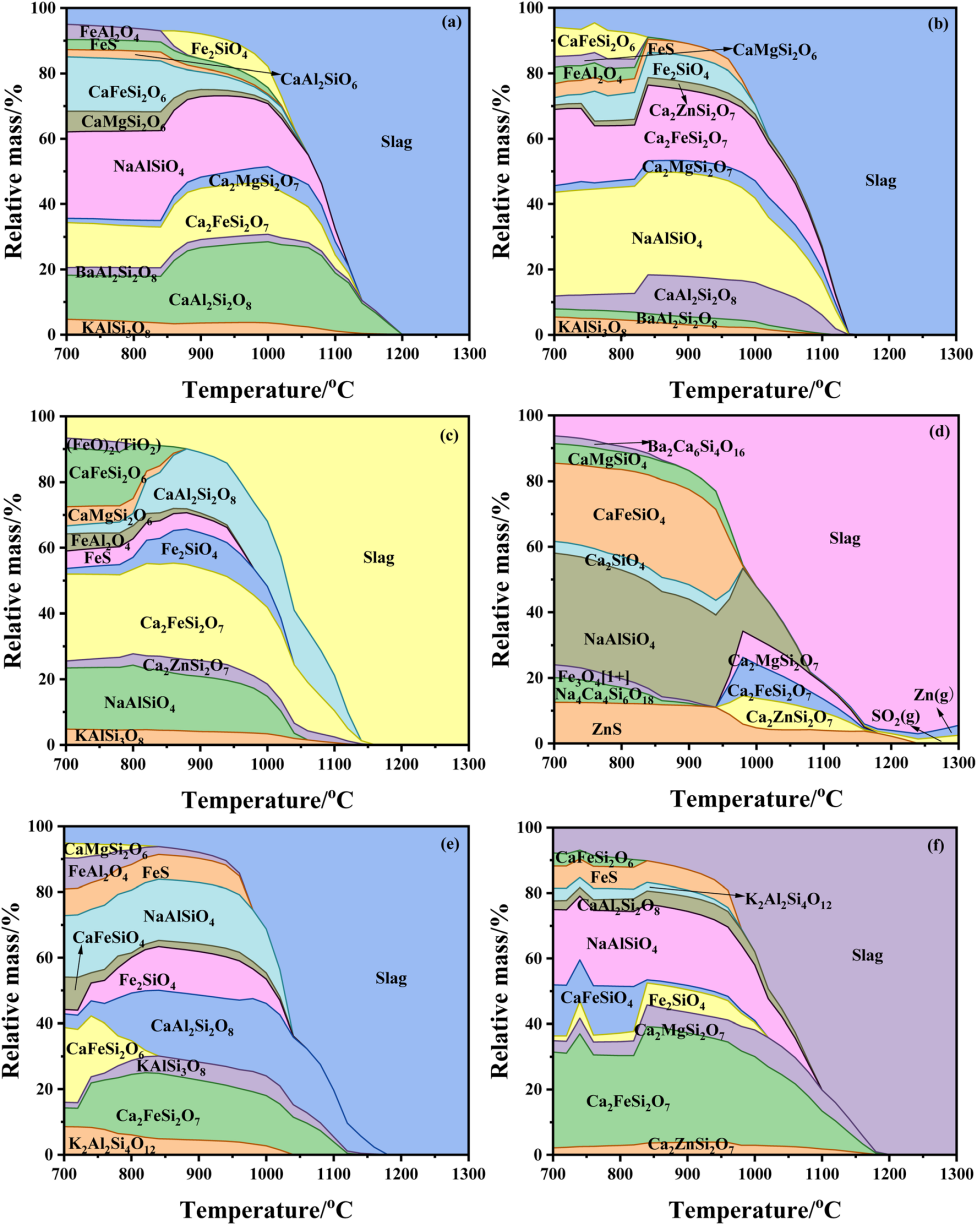
Mineral evolution of ash slag at different positions of the RSC: (a) radiation screen top 25 m; (b) 25 m around spray water; (c) 20 m around spray water; (d) 15 m around spray water; (e) floating ash on water-cooled wall; (f) boiler bottom 0–1 m.

The mineral evolution of ash slag from different regions of the RSC at 700–1300 °C under a weak reducing atmosphere was calculated using FactSage thermodynamic software, and the results are shown in Fig. 8. Among them, Fig. 8(a) displays the mineral evolution results in ash slag at the top 25 m of the radiation screen during the heating process. It can be seen that its main minerals were augite (CaFeSi_2_O_6_ and CaMgSi_2_O_6_), nepheline (NaAlSiO_4_), melilite (Ca_2_MgSi_2_O_7_ and Ca_2_FeSi_2_O_7_), and anorthite (CaAl_2_Si_2_O_8_), and as the temperature increased, they gradually melted and entered the liquid-phase slag. Eventually, at about 1200 °C, all the minerals were melted and transformed into liquid-phase slag. In addition, due to the weak reducing atmosphere, Fe_2_O_3_ in ash slag was reduced to FeO, which underwent a further low-temperature eutectic reaction with SiO_2_ and Al_2_O_3_ to form hercynite (FeAl_2_O_4_) and fayalite (Fe_2_SiO_4_). At 700–1050 °C, FeO also reacted with sulfur to form a small amount of pyrite (FeS), while there was also a small amount of microcline (KAlSi_3_O_8_) in the low-temperature region, which was consistent with the mineral composition detected by XRD. The chemical reactions involved above are shown in eqn (1)–(10).^28–32^Fig. 8(b) shows the mineral evolution results of ash slag at 25 m around the spray water, and it can be seen that the main minerals were similar to those of Fig. 8(a), with melilite (Ca_2_ZnSi_2_O_7_, Ca_2_MgSi_2_O_7_, and Ca_2_FeSi_2_O_7_), nepheline (NaAlSiO_4_), and anorthite. However, compared with Fig. 8(a), the content of anorthite in the ash slag at 25 m around the spray water significantly reduced, while the content of nepheline and Ca_2_FeSi_2_O_7_ significantly increased, and the minerals melt and transform into liquid-phase slag at lower temperatures (∼1140 °C). Fig. 8(c) presents the mineral evolution results in the ash slag at 20 m around the spray water, which is similar to Fig. 8(a and b), with the main minerals being melilite, anorthite, and nepheline, but the content of the crystalline minerals in them was significantly lower than that of Fig. 8(a and b), especially the content of nepheline. In contrast, the content of iron-bearing minerals significantly increased, although FeS also appeared at 700–1050 °C in Fig. 8(c), and the contents of hercynite and fayalite almost remained unchanged. From the above results, it can be seen that the mineral composition of the ash slag was similar at the top of the RSC, *i.e.*, 25 m at the top of the radiation screen and 25/20 m around the spray water. Combining the mineral results of FactSage thermodynamic calculations with the XRD results indicated that there was a certain spatial distribution pattern of ash slag deposition behavior in the RSC.

Fig. 8(d) exhibits the mineral evolution results in the ash slag at 15 m around the spray water, and the main minerals were olivine (CaFeSiO_4_), nepheline, and melilite, where the formation of olivine (CaFeSiO_4_) is shown in eqn (11). Unlike Fig. 8(a–c), the ash slag at 15 m around the spray water had a higher content of nepheline, which was attributed to its ash chemical composition. According to the ash chemical composition in Table 5, the ash slag at 15 m around the spray water had the highest Na_2_O content, indicating that Na_2_O predominantly existed in the form of nepheline and was deposited around the water-cooled wall. Meanwhile, due to the high Zn content, ZnS appeared in the low-temperature region (700–1240 °C), whereas at 950 °C, the ZnS content decreased, but Zn-bearing Ca–Zn feldspar (Ca_2_ZnSi_2_O_7_) was formed. As the temperature increased (>1150 °C), gaseous Zn appeared, as well as gaseous SO_2_ in the high-temperature region due to the higher S content. Thus, due to the presence of refractory material Ca_2_ZnSi_2_O_7_ in the high-temperature region in Fig. 8(d), its liquid-phase temperature was higher than that of Fig. 8(a–c), exceeding 1300 °C. Fig. 8(e) shows the mineral evolution results in the floating ash of the water-cooled wall, and its main minerals were still nepheline, anorthite, and melilite. At 700–850 °C, the content of Ca–Fe augite (CaFeSi_2_O_6_) was significantly higher, while the content of anorthite was lower. As the temperature increased, the contents of both anorthite and olivine significantly increased, while CaFeSi_2_O_6_ disappeared; the reaction is shown in eqn (11). Fig. 8(f) shows the mineral evolution results in the ash slag from 0–1 m at the bottom of the boiler, where the main minerals were nepheline and Ca–Fe yellow feldspar (CaFeSi_2_O_7_). At 700–850 °C, the content of Ca–Fe augite (CaFeSi_2_O_6_) was low, but the content of Ca–Fe olivine (CaFeSiO_4_) was high. As the temperature increased (850–1020 °C), the content of CaFeSiO_4_ decreased, but the content of iron olivine (Fe_2_SiO_4_) significantly increased, and it was inferred that the ferrous ions replaced the calcium ions in CaFeSiO_4_ to form Fe_2_SiO_4_; the reaction is shown in eqn (12). In addition, according to the ash chemical analysis in Table 5 and it was found that the ash slag contained toxic and harmful trace elements, such as Ba and Zn, which may pose a potential threat to the environment. According to the above figures, it was found that there were silicate minerals containing Ba and Zn in the ash slag, and the sulfur in the coal ash was also present in the form of FeS and ZnS.

1 Al_2_O_3_ + FeO → FeAl_2_O_4_

2 SiO_2_ + 2FeO → Fe_2_SiO_4_

3 CaO + FeO + 2SiO_2_ → CaFeSi_2_O_6_

4 CaO + MgO + 2SiO_2_ → CaMgSi_2_O_6_

5 Na_2_O + Al_2_O_3_ + 2SiO_2_ → 2NaAlSiO_4_

6 2CaO + 2SiO_2_ + MgO → Ca_2_MgSi_2_O_7_

7 2CaO + 2SiO_2_ + FeO → Ca_2_FeSi_2_O_7_

8 CaO + SiO _2_+ Al_2_O_3_ → CaAl_2_Si_2_O_8_

9 K_2_O + Al_2_O_3_ + 2SiO_2_ → 2KAlSi_3_O_8_

10 CaO + FeO + SiO_2_ → CaFeSiO_4_

11 2CaFeSi_2_O_6_ + Al_2_O_3_ + 3SiO_2_ → 2CaAl_2_Si_2_O_8_ + Fe_2_SiO_4_

12 2CaFeSiO_4_ + FeO + SiO_2_ → Fe_2_SiO_4_ + Ca_2_FeSi_2_O_7_

### 3.4 *In situ* observation using HTSOM

#### 3.4.1 Heating and melting process

**Fig. 9:**
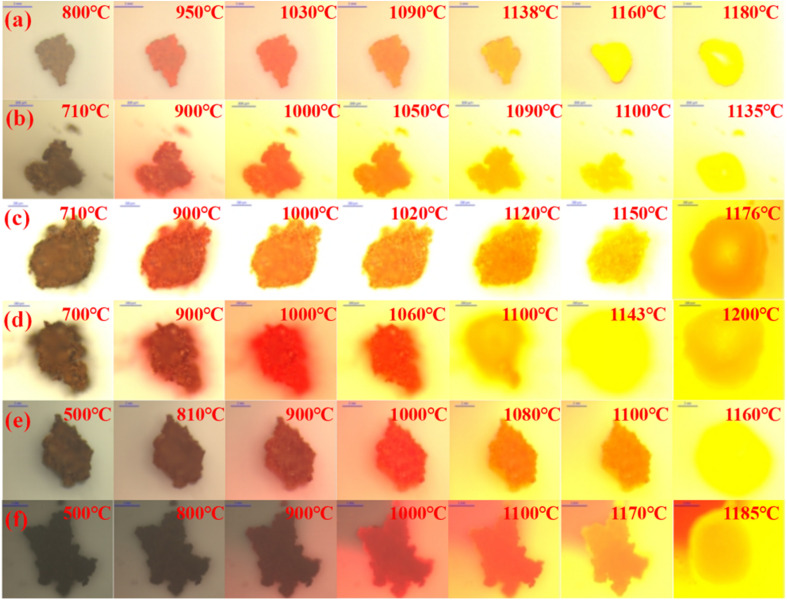
*In situ* melting observation of ash slag at different positions of the RSC during the heating process: (a) radiation screen top 25 m; (b) 25 m around spray water; (c) 20 m around spray water; (d) 15 m around spray water; (e) floating ash on water-cooled wall; (f) boiler bottom 0–1 m.

The heating–melting process and cooling crystallization process of ash slag at different areas of the RSC were observed *in situ* using HTSOM. Among them, the heating–melting process of ash slags in different key areas is shown in Fig. 9. From the overall observation of the changes, it was found that with the increase in temperature, the ash slag samples first exhibited obvious volume shrinkage behavior, and with the increase in temperature, the interior of the samples collapsed and rapidly contracted. When the volume shrank to the minimum, the samples began to melt, and a distinct red molten substance could be observed, exhibiting rapid melting from the periphery to the interior. The increase in red molten material accelerated the melting process of the internal ash slag, and within a short period of time, the ash slag completely melted into a liquid phase with strong fluidity and flowed in all directions. All ash slag samples underwent three stages of “shrinkage–melting–flow”. The last picture during the melting process of each sample in Fig. 9(a–f) represents the maximum degree of melt flow extension; that is, as the temperature increased, the liquid-phase slag no longer flowed and extended. It can be seen that the complete melting flow temperature in Fig. 9(d) was 1200 °C, higher than that of other samples, which may be related to the unique ash chemical composition at 15 m around the spray water. As can be seen from Table 5, the S, Na, and Zn contents of the ash slag were higher at 15 m around the spray water. Combined with the mineral evolution results in Fig. 9(d), it can be observed that, although Na reacted with other oxides in low-temperature eutectic reactions and generated low melting point minerals (nepheline NaAlSiO_4_ and Na_4_Ca_4_Si_6_O_18_), the higher content of Zn would react with other oxides to form refractory mineral Ca_2_ZnSi_2_O_7_, which was the main reason for the melting delay and reduced fluidity in Fig. 9(d).

#### 3.4.2 Cooling crystallization process

**Fig. 10:**
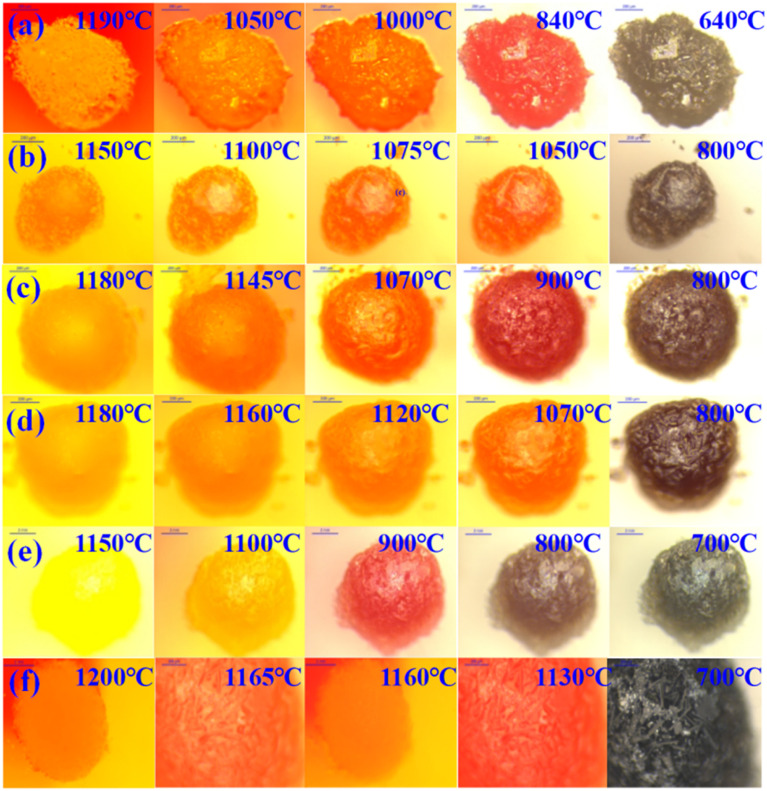
*In situ* crystallization observation of ash slag at different positions of the RSC during the cooling process: (a) radiation screen top 25 m; (b) 25 m around spray water; (c) 20 m around spray water; (d) 15 m around spray water; (e) floating ash on water-cooled wall; (f) boiler bottom 0–1 m.

Fig. 10 illustrates the *in situ* crystallization observation results of ash slag samples from different regions in the RSC during the cooling process. At the high-temperature stage, the minerals in the ash slag began to form crystal nuclei and initially grew. As the temperature decreased, the crystal size continued to increase until it reached complete crystallization. The last picture of each sample cooling process in Fig. 10(a–f) indicates complete crystallization, *i.e.*, the crystal stopped growing, after which the crystal morphology did not change. From observation of the overall changes, it could be seen that the ash slag samples were mainly precipitated as interlaced rod-like crystals and a small number of square crystals with different sizes during the cooling process, among which the square crystals in Fig. 10(a) had larger sizes.

## 4. Conclusions

(1) Through elemental analysis, it could be concluded that due to insufficient temperature, some carbon particles in the fly ash inside the RSC failed to gasify, resulting in a high carbon content in the floating ash of the water-cooled wall. The sulfur element was mainly enriched in the middle area of the RSC.

(2) The mineral evolution results calculated with FactSage thermodynamic software showed that the high content of alkaline oxides CaO, Fe_2_O_3_, and Na_2_O in the ash slag of different areas in the RSC will undergo low-temperature eutectic reactions with acidic oxides SiO_2_ and Al_2_O_3_, forming low melting point minerals, such as nepheline, anorthite, augite, and melilite. However, the ash slag at 15 m around the spray water contained a high content of ZnO, which could react with CaO and SiO_2_ to form the refractory mineral Ca–Zn feldspar (Ca_2_ZnSi_2_O_7_).

(3) From *in situ* observation, it was found that the ash slag samples exhibited significant shrinkage behavior during the initial heating stage and then entered the melting stage after the volume shrank to a minimum, showing an expansion from the periphery to the interior, ultimately forming a liquid-phase slag with strong fluidity. All the ash slag samples underwent three stages of “shrinkage–melting–flow”. Due to the formation of refractory mineral Ca_2_ZnSi_2_O_7_ during heating of the ash slag at 15 m around the spray water, resulting in a higher melting temperature, which required a higher temperature to achieve full flow. During the cooling process, interlaced rod-like crystals and a small number of square crystals of different sizes were mainly precipitated.

## Author contributions

Chunlei Gao: investigation, data curation, formal analysis, writing – original draft. Linmin Zhang: supervision, writing – review & editing, project administration. Xudong Song: supervision, writing – review & editing, project administration. Jingyuan Cao: data curation, investigation. Qinghua Guo: data curation, investigation. Guangsuo Yu: writing – review & editing, resources, supervision, project administration.

## Conflicts of interest

There are no conflicts to declare.

## Data Availability

The data used to support the results of this work are available from the corresponding author upon request.
